# Integrin activating molecule-talin1 promotes skin fibrosis in systemic sclerosis

**DOI:** 10.3389/fimmu.2024.1400819

**Published:** 2024-05-28

**Authors:** Dan Xu, Xiandun Yuan, Zhaohua Li, Rong Mu

**Affiliations:** Department of Rheumatology and Immunology, Peking University Third Hospital, Beijing, China

**Keywords:** systemic sclerosis, fibrosis, integrin activating molecules, talin1, integrin

## Abstract

**Background:**

Integrin-dependent cell adhesion and migration play important roles in systemic sclerosis (SSc). The roles of integrin activating molecules including talins and kindlins, however, are unclear in SSc.

**Objectives:**

We aimed to explore the function of integrin activating molecules in SSc.

**Methods:**

Transcriptome analysis of skin datasets of SSc patients was performed to explore the function of integrin-activating molecules including talin1, talin2, kindlin1, kindlin2 and kindlin3 in SSc. Expression of talin1 in skin tissue was assessed by multiplex immunohistochemistry staining. Levels of talin1 in serum were determined by ELISA. The effects of talin1 inhibition were analyzed in human dermal fibroblasts by real-time PCR, western blot and flow cytometry.

**Results:**

We identified that talin1 appeared to be the primary integrin activating molecule involved in skin fibrosis of SSc. Talin1 was significantly upregulated and positively correlates with the modified Rodnan skin thickness score (mRSS) and the expression of pro-fibrotic biomarkers in the skin lesions of SSc patients. Further analyses revealed that talin1 is predominantly expressed in the dermal fibroblasts of SSc skin and promotes fibroblast activation and collagen production. Additionally, talin1 primarily exerts its effects through integrin β1 and β5 in SSc.

**Conclusions:**

Overexpressed talin1 is participated in skin fibrosis of SSc, and talin1 appears to be a potential new therapeutic target for SSc.

## 1 Introduction

Systemic sclerosis (SSc) is a rare autoimmune disease characterized by excessive fibrosis of skin and internal organs, burdened by the highest mortality among rheumatic diseases (1, 2). Unfortunately, the treatment of skin fibrosis still poses a significant challenge due to the limited therapeutic effects, potentially hazardous side effects, and varying degrees of recurrence (3, 4). Integrins, crucial cell adhesion and signaling proteins, play vital roles in a wide range of biological functions. They are transmembrane heterodimers composed of a ligand-specific α subunit in noncovalent association with a β subunit. Integrins facilitate bidirectional signaling between the extracellular and intracellular compartments, as their intracellular domains are linked to the actomyosin cytoskeleton, while their extracellular domains bind to extracellular matrix (ECM) ligands (5). Emerging evidence has shown that integrins play key roles in the pathogenesis of SSc. Gerber et al. found that skin fibrosis in the SSc model could be prevented by modulating integrin β1 or β3 (6). Targeting integrin αv with neutralizing antibodies or the small-molecule inhibitor CWHM12 significantly mitigates bleomycin-induced fibrosis in mouse skin and lungs (7–9). The dual integrin αvβ3/αvβ5 inhibitor cilengitide blocked skin and lung fibrosis when therapeutically administered in a murine model of SSc (10). However, current integrin-targeted therapies in SSc have yet to demonstrate substantial clinical efficacy (11, 12), potentially due to the activation states of integrins.

Integrins, which can exist in both activated and inactivated states, display varying affinities for ligands, enabling them to modulate cellular responses to changes in the extracellular environment (13). Under pathological conditions, however, this modulation may be excessively amplified (14, 15). Antagonistic molecules that bind to integrins, preventing endogenous ligand interaction, can inadvertently activate integrins, leading to inadequate clinical outcomes or safety concerns. This phenomenon has been observed with the integrin αIIbβ3-targeting drug eptifibatide, which induced severe thrombocytopenia in some patients (16). Thus, a deeper understanding of the underlying mechanisms of integrin activation in SSc is pivotal.

Talins and kindlins are essential activating molecules of integrin signaling and required for evoking a conformational change that shifts the integrin into a high-affinity state (17, 18). Talin, a cytosolic protein found in high concentrations in focal adhesion, is a large protein (270 kDa) that links integrins to the actin cytoskeleton (19, 20). Mammals express two talin paralogues, talin1 and talin2, which share 80% homology (21). While talin1 is ubiquitously expressed, talin2 is enriched in muscle and neuronal tissues (22, 23). Kindlin, on the other hand, is a 78 kDa cytosolic protein that directly interacts with integrins and is essential for the proper assembly of focal adhesion (24). There are three kindlin subtypes, kindlin1-3, with varying expression patterns. Kindlin1 is mainly expressed in epithelial cells, while kindlin2 is ubiquitously expressed outside the haematopoietic system. In contrast, kindlin3 is restricted to the haematopoietic system (25). By binding to the cytosolic tail of integrin β subunits, talins and kindlins might activate integrins, increasing affinity for ligands, thus promoting cell migration and ECM assembly and remodeling (26). These findings suggest that talins and kindlins may be involved in the development of tissue fibrosis by regulating integrin activation. However, to our knowledge, the role of talins and kindlins in SSc has never been reported, nor has the underlying mechanism.

Here, we systematically combined bioinformatic methods with experimental techniques to evaluate integrin activating molecules including talin1, talin2, kindlin1, kindlin2 and kindlin3 in SSc. Our study found the increased expression of talin1 in skin lesions and serum of SSc patients, identified the role of talin1 in skin fibrosis and revealed the molecular mechanism of talin1 in skin fibrosis. This study will elaborate our understanding of integrin activating molecules promoting progression of SSc as well as provide potential therapeutic targets for SSc.

## 2 Methods

### 2.1 Patients and clinical assessment

We obtained skin samples from 28 patients with SSc and serum samples from 84 patients with SSc. All patients fulfilled the criteria for SSc according to the 2013 American College of Rheumatology/European League Against Rheumatism classification criteria and were grouped according to the classification system proposed by LeRoy et al. Skin and serum samples were also collected from 8 health controls (HCs) and 40 age- and sex-matched HCs, respectively. All serum samples were stored at -80°C before use. The study was approved by the Ethical Committee of the Peking University Third Hospital. Written informed consents were obtained before patients and HCs were entered into this study, according to the Declaration of Helsinki.

### 2.2 Multiplex immunohistochemistry staining

mIHC was performed using an Opal Multiplex mIHC kit (Akoya Biosciences, USA). FFPE tissue sections were incubated with primary antibodies for vimentin (1:1000, Abcam, UK), talin1 (1:800, Abcam, UK), α-SMA (1:500, Cell Signaling Technology, USA), ITGB1 (1:400, Cell Signaling Technology, USA) and ITGB5 (1:400, Cell Signaling Technology, USA) for 45 minutes. Then, these slides were incubated with polymeric HRP-conjugated secondary antibodies (Zhongshanjinqiao, China) and followed by incubation with Opal fluorophore-conjugated tyramide signal amplification (TSA) (Akoya Biosciences, USA) at a 1:100 dilution. Nuclei were counterstained using DAPI. Images were captured for each case under a Vectra Polaris multispectral imaging system and analyzed by QuPath software (version 0.4.3).

### 2.3 Measurement of serum levels of talin1

Serum talin1 levels were measured with a specific ELISA kit (Quantikine ELISA Human talin1 Immunoassay; FineTest, China). The procedures were performed according to the manufacturer’s instructions.

### 2.4 Cell culture and treatment

The isolated human skin fibroblasts were cultured in Dulbecco’s Modified Eagle’s Medium (DMEM) (Invitrogen, CA, USA). For RNA interference experiment, cells were transfected with talin1 siRNA (100 pmol) or control siRNA (100 pmol) using Lipofectamine 2000 (Invitrogen, USA). The siRNA for negative control (NC) and talin1 were purchased (Shengong, Shanghai, China) and diluted in RNase-free double-distilled water. Sequences for talin1 siRNA are as follows: talin1-siRNA forward 5’-CCUUCGUGGAUUACCAAACAATT-3’, and reverse 5’-UUGUUUGGUAAUCC ACGAAGGTT-3’. After 48 h, the treated cells were collected for further experiments. In addition, the Cell Counting Kit 8 (DOJINDO, Japan) assay was employed to evaluate cell viability and proliferation.

### 2.5 Migration assay

Cells with or without transfection were plated into 12-well plates. After reaching 100% confluence, the cells were then washed with PBS and wounded by scraping with a 200μl pipette tip. After washing with PBS, the cells were then incubated in DMEM without FBS. Photographs of the wound area were taken at 0 and 24 hours.

### 2.6 Apoptosis assay

The Annexin V Apoptosis Detection Kit (BioGems, USA) was utilized to assess cellular apoptosis. Cells with or without transfection were washed, centrifuged, and then resuspended in PBS buffer containing annexin V-FITC. The cells were incubated at room temperature for 15 minutes. The samples were analyzed with a CytoFLEX flow cytometer (Beckman Coulter, USA).

### 2.7 RNA isolation, reverse transcription, and real-time RT-PCR

Total RNA samples were extracted from cells using TRIzol (Invitrogen, USA) according to the manufacturer’s instructions. Complementary DNA (cDNA) was synthesized using High-Capacity cDNA Reverse Transcription Kit (Applied Biosystems, USA). Real-time PCR was performed with SYBR Green PCR Kit (Applied Biosystems, USA) and analyzed with an ABI Prism 7900 Detector System (Applied Biosystems, USA). The Real-time RT-PCR primers are listed in **Supplementary Table S1**.

### 2.8 Western blot analysis

Proteins were separated by SDS-PAGE and transferred to a polyvinylidene difluoride membrane. After blocking with 5% BSA, blotted proteins were incubated with antibodies against talin1 (1:1000, Abcam, UK), α-SMA (1:1000, Cell Signaling Technology, USA), col1a1 (1:1000, Abcam, UK) primary antibody and HRP-conjugated secondary antibodies (Servicebio, China). Then, the membranes were visualized with an enhanced chemiluminescence system (Thermo Fisher Scientific Inc., USA). The intensity of bands was quantified using ImageJ software.

### 2.9 Bioinformatic analysis

Microarray dataset (accession number GSE58095) and RNA-seq dataset (accession number GSE130955) from NCBI Gene Expression Omnibus (GEO) (https://www.ncbi.nlm.nih.gov/geo/) are available. The differential mRNA expression analysis of integrin activating molecules was performed using the limma package. The violin plot was also utilized to visualize the expression of integrin activating molecules. The correlations between mRNA expression of integrin activating molecules and clinical characteristic were analyzed.

### 2.10 Statistical analysis

Statistical analysis was carried out with Student’s t*-*test or Mann-Whitney test for comparing the mean or median, respectively. Chi-squared test and Fisher’s exact probability test were performed for the independence between the two groups in terms of type and clinical data of SSc patients. *P*<0.05 was considered statistically significant.

## 3 Results

### 3.1 The expression of talin1 increased in the skin tissues of patients with SSc

**Figure 1:**
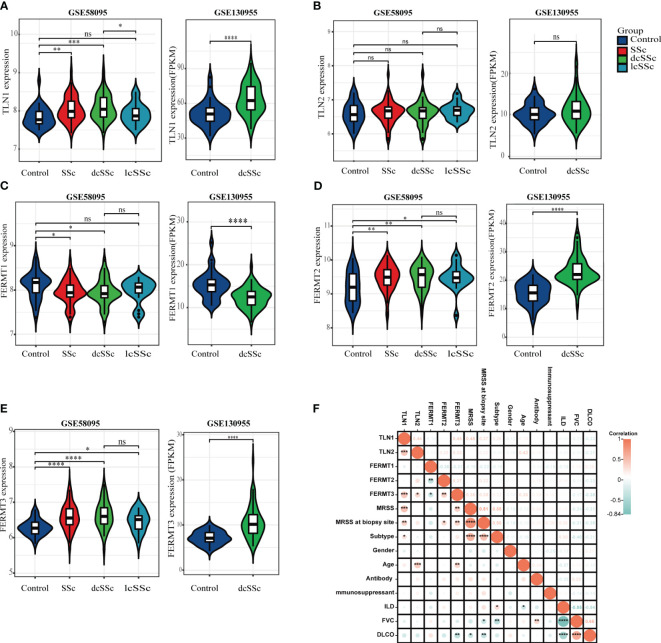
Transcriptome analysis reveals enhanced talin1 expression among integrin activating molecules in the skin tissues of patients with SSc. **(A)** mRNA expression levels of talin1 between SSc and HCs. **(B)** mRNA expression levels of talin2 between SSc and HCs. **(C)** mRNA expression levels of kindlin1 between SSc and HCs. **(D)** mRNA expression levels of kindlin2 between SSc and HCs. **(E)** mRNA expression levels of kindlin3 between SSc and HCs. **(F)** Correlation between expression of integrin activating molecules with clinical characteristics in GEO database. * indicates P<0.05; ** indicates P<0.01; *** indicates P<0.001; **** indicates P<0.0001; ns indicates P>0.05.

The transcriptome analysis was performed using the microarray dataset (GSE58095, 61 SSc versus 36 HCs) and RNA-seq dataset (GSE130955, 48 SSc versus 33 HCs) from GEO database, and found that the mRNA expression levels of talin1 were significantly higher in skin samples from SSc patients than in those from HCs (*P*<0.001). Concerning the SSc subgroups, the mRNA expression levels of talin1 were higher in dcSSc than in lcSSc (*P*=0.043) (**Figure 1A**). Further exploration of the clinical value revealed that the expression of talin1 was positively correlated with modified Rodnan skin thickness score (mRSS) (r=0.554, *P*<0.001) and mRSS at the biopsy site (r=0.37, *P*<0.01) (**Figure 1F**).

**Figure 2:**
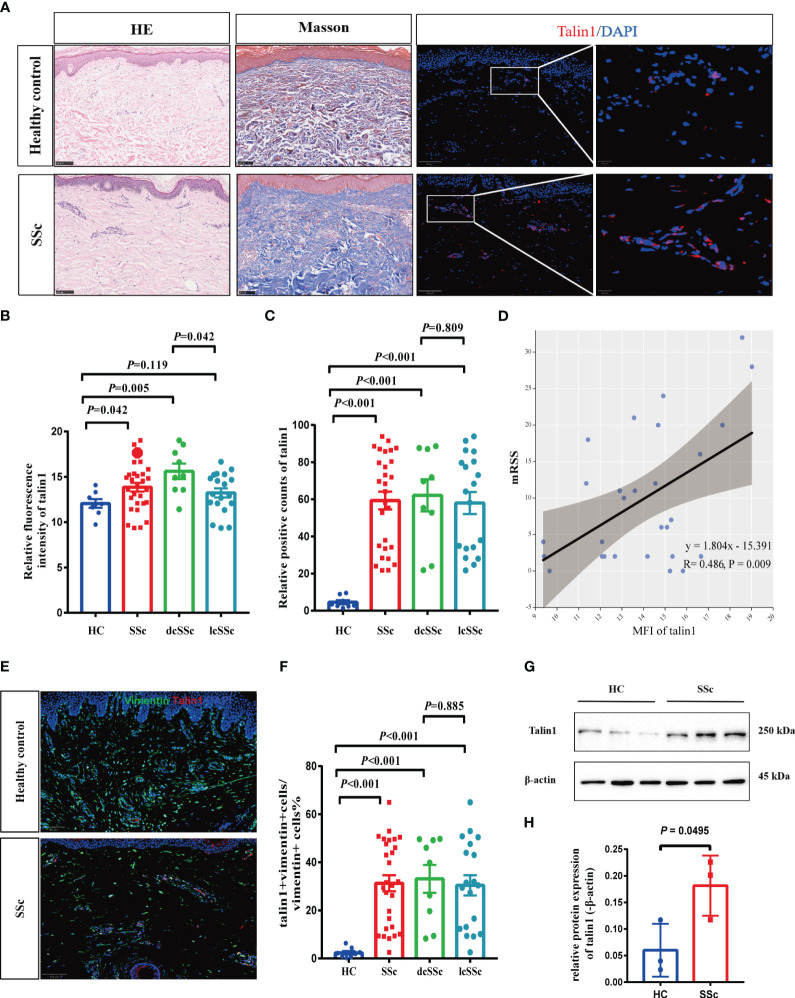
Talin1 is highly expressed in skin fibroblasts in SSc. **(A)** HE, Masson trichrome, and immunofluorescence staining of skin tissues between SSc and HCs. **(B)** The MFI of talin1 between SSc and HCs. **(C)** The percentage of talin1 positive cells between SSc and HCs. **(D)** MFI of talin1 was positively correlated with mRSS in SSc. **(E)** Double immunofluorescence staining talin1 and vimentin in SSc and HCs. **(F)** The percentage of talin1-positive fibroblasts in SSc and HCs. **(G)** Relative protein levels of talin1 in the skin fibroblast between SSc and HCs. **(H)** Semi-quantification of the western blot results by ImageJ software.

To verify this finding, we examined talin1 expression in an independent sample group from the Chinese population consisting of 28 SSc patients and 8 HCs. The clinical characteristics are shown in **Table 1**. More talin1-positive cells were observed in the skin of SSc patients than in the skin of HCs (*P*<0.001), especially in dcSSc (**Figures 2A, B**). The mean fluorescence intensity (MFI) of talin1 is higher in the skin of SSc patients than in the skin of HCs (*P*=0.042) (**Figure 2C**). The MFI of talin1 was positively correlated with mRSS in SSc (r=0.486, *P*=0.009) (**Figure 2D**).

**Table 1:** Clinical characteristics of patients with SSc.

Characteristics	SSc (n=28)
Demographic characteristics	
Sex, female/male,	25/3
Age, years	53.7 ± 12.8
Disease duration, months	42.8 ± 59.1
Limited/diffuse SSc	18/10
mRSS	9.9 ± 9.2
Clinical symptoms	
Raynaud’s phenomenon	24 (85.7%)
Digital ulcer (%)	2 (7.1%)
Joint involvement	3 (10.3%)
Interstitial lung fibrosis (%)	16 (57.1%)
PAH (%)	3 (10.3%)
Cardiac involvement (%)	2 (7.1%)
GI involvement (%)	16 (57.1%)
Renal crisis (%)	0 (0)
Autoantbodies	
ANA (+)	27 (96.4%)
ACA (+)	13 (46.4%)
Anti-Scl-70 (+)	9 (32.1%)
Anti-RNAP III (+)	1 (3.6%)
Treatment	
Prednisone	5 (%)
CTX	1 (3.6%)
MMF	1 (3.6%)
AZA	1 (3.6%)
Treatment naive	20 (71.4%)

SSc, systemic sclerosis; IPF, interstitial pulmonary fibrosis; PAH, pulmonary arterial hypertension; GI, gastrointestinal involvement.ANA, anti-nuclear antibody; ACA, anti-centromere antibody; anti-Scl-70, anti-topoisomerase I antibody; anti-RNAP III, anti-RNA polymerase III antibody; CTX, cyclophosphamide; MMF, mycophenolate mofetil; AZA, azathioprine. Values are given as mean ± S.D. or unless stated otherwise.The symbol (+) indicates that the antibody is positive.

We also observed that the expression levels of kindlin1 was significantly reduced (**Figure 1C**). In addition, the mRNA expression levels of kindlin2 and kindlin3 were significantly higher in skin samples from SSc patients than in those from HCs, whereas were comparable between dcSSc and lcSSc ((**Figures 1D, E**). There was no correlation between the expression of kindlin1, kindlin2 and mRSS (**Figure 1F**). The expression of kindlin3 exhibited a modest positive correlation with mRSS (r=0.33, *P*<0.05) (**Figure 1F**). There was no significant difference on the mRNA expression levels of talin2 between SSc and HCs (**Figure 1B**).

### 3.2 Talin1 is highly expressed in skin fibroblasts in SSc

We performed a double immunofluorescence staining and found that talin1 was expressed in fibroblasts (vimentin marked) (**Figure 2E**). In SSc, 31.3% (± 17.8%) of vimentin-positive fibroblasts were stained for talin1, whereas only 2.4% (± 2.1%) of fibroblasts in healthy skin expressed talin1 (*P*<0.001) (**Figure 2F**). Furthermore, we isolated the fibroblast from SSc and healthy skin and showed that level of talin1 is higher in SSc skin fibroblast than in healthy control by western blot analysis (**Figures 2G, H**).

### 3.3 The levels of talin1 in serum were increased in the patients with SSc

The concentrations of talin1 in the serum of 84 SSc patients and 40 HCs were measured. The levels of talin1 in the serum of patients with SSc were higher than those of HCs (2.018 ± 0.945ng/ml *vs* 1.069 ± 0.582 ng/ml, *P*<0.0001). We defined talin1 concentration greater than the mean + 2SD of the value in HCs (2.234ng/mL) as high talin1, which was found in 19 SSc patients. Patients with diffuse type of SSc, complications of SRC and elevated ESR had statistically significantly higher serum talin1 (**Table 2**). Accordingly, the concentrations of talin1 in patients with dcSSc (2.280 ± 1.067ng/mL) were significantly higher than in patients with lcSSc (1.818 ± 0.752ng/mL, *P*=0.025). Moreover, the concentrations of talin1 in the SSc patients with SRC were significantly higher than in SSc patients without SRC (2.889 ± 1.059 *vs* 1.939 ± 0.901ng/mL *P*=0.020). There was also a trend towards increased percentage of IPF in SSc patients with high serum talin1 level than those with normal serum talin1 level (*P*=0.083). Consistently, the levels of talin1 in serum were significantly increased in SSc patients with IPF than in SSc patients without IPF (2.226 ± 1.025 vs 1.603 ± 0.584ng/mL *P*=0.003). There were no statistically significant differences between sex, age at onset, disease duration, other clinical symptoms.

**Table 2:** Correlation of serum soluble talin1 levels with clinical features and symptoms in SSc patients.

	SSc with elevated talin1 levels (n=19)	SSc with normal talin1 levels (n=65)	*P*
Demographic characteristics			
Sex, male/female	2/17	6/59	0.866
Age, years	54 (47-58)	59 (47-65)	0.200
Disease duration, months	103 (48-168)	96 (42-204)	0.865
Subtypes of SSc Diffuse/limited SSc	14/5	28/37	0.019*
Clinical symptoms			
Raynaud’s phenomenon	15/4	51/14	0.847
Digital ulcer	4/15	10/55	0.562
Joint involvement	7/12	23/42	0.979
IPF	16/3	41/24	0.083
PAH	4/15	12/53	0.751
Cardiac involvement	0/19	12/53	0.06
Gastrointestinal involvement	8/11	32/33	0.507
Renal crisis	4/15	3/62	0.043*
Laboratory characteristics			
ANA (+/-)	16/3	64/1	0.035*
Anti-RNP	5/14	64, 9/55	0.293
Anti-CENPA (+/-)	18, 5/13	64, 24/40	0.446
Anti-CENPB (+/-)	18, 5/13	60, 24/38	0.396
Anti-Scl-70 (+/-)	18, 6/12	65, 16/49	0.548
Anti-RNAP III (+/-)	18, 2/16	64, 4/60	0.608
WBC	6.50 (5.30-7.50)	5.30 (4.15-6.95)	0.103
ESR	23 (11-51)	13 (9-22)	0.02*
Immunoglobulin A	2.36 (1.44-3.82)	2.17 (1.48-3.07)	0.653
Immunoglobulin G	11.50 (9.60-15.90)	12.70 (9.95-15.45)	0.474
Immunoglobulin M	1.14 (0.62-1.70)	0.93 (0.68-1.20)	0.185
C3	0.95 (0.80-1.17)	0.89 (0.76-1.00)	0.233
C4	0.23 (0.17-0.25)	0.20 (0.15-0.23)	0.126
FVC, % of predicted	10, 86.70 (69.67-104.98)	39, 80.20 (68.20-94.00)	0.264
DLCO, % of predicted	10, 57.45 (35.83-72.21)	39, 53.90 (44.60-62.00)	0.893
FVC/FEV1	10, 86.27 (78.56-87.88)	39, 83.15 (77.43-85.82)	0.244

SSc, systemic sclerosis; IPF, interstitial pulmonary fibrosis; PAH, pulmonary arterial hypertension; ANA, anti-nuclear antibody; anti-CENPA/B, anti-centromere A/B antibody; anti-RNP, anti-U1 RNP antibody; anti-Scl-70, anti-topoisomerase I antibody; anti-RNAP III, anti-RNA polymerase III antibody; WBC, white blood cell; ESR, erythrocyte sedimentation rate; FVC, forced vital capacity; DLCO, carbon monoxide breath diffusion capacity; FEV1, forced expiratory volume in one second. Data in the table are presented as percentages or medians (25th-75th percentiles).The symbol (+/-) indicates that the antibody is positive or negative; * indicates P<0.05.

### 3.4 Talin1 promotes the activation and migration of skin fibroblasts

**Figure 3:**
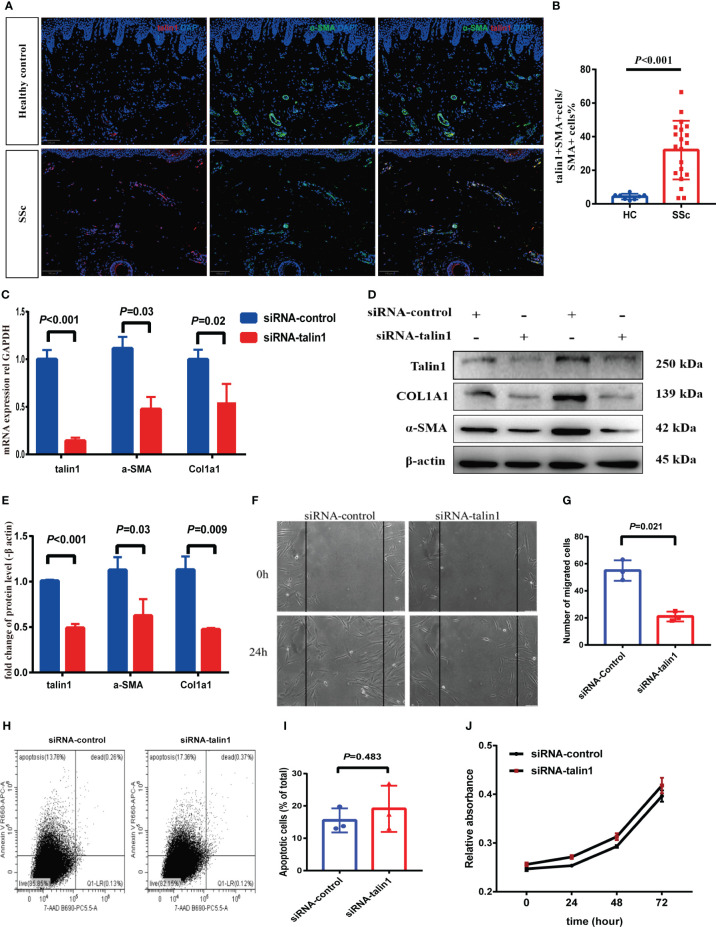
Talin1 promotes the activation and migration of skin fibroblasts. **(A, B)** Expression and quantification of talin1 in α-SMA positive cells in the skin of SSc and HCs. **(C)** Relative transcript levels of talin1, α-SMA and COL1A1 measured by real-time PCR in SSc patients’ fibroblasts with or without talin1 knockdown. **(D)** Relative protein levels of talin1, α-SMA and COL1A1 in SSc patients’ fibroblasts with or without talin1 knockdown. **(E)** Semi-quantification of the western blot results by ImageJ. **(F)** Scratch assay was performed on primary fibroblast treated with or without talin1 siRNA. **(G)** Quantification of migrated SSc patients’ fibroblasts with or without talin1 knockdown. **(H)** Detection of apoptosis and necrosis using Annexin V-APC and 7AAD dual staining on SSc patients’ fibroblasts with or without talin1 knockdown. **(I)** Bar graph for apoptotic cell percentages. **(J)** CCK-8 cell viability assay of SSc patients’ fibroblasts with or without talin1 knockdown.

In skin tissue, the percentage of talin1-positive cells co-expressing α-SMA increased in SSc compared to HC (**Figures 3A, B**). Talin1 was knocked down in SSc primary skin fibroblasts to explore its roles in fibroblasts. The mRNA and protein levels of col1a1 and α-SMA decreased in SSc primary fibroblasts after talin1 knockdown treatment (**Figures 3C–E**). The migratory ability of SSc primary fibroblasts weakened after knocking down talin1 (**Figures 3F, G**), whereas the cell apoptosis (**Figures 3H, I**) and proliferation (**Figure 3J**) remains unchanged.

### 3.5 Talin 1 activated the integrin family signaling pathway in SSc

**Figure 4:**
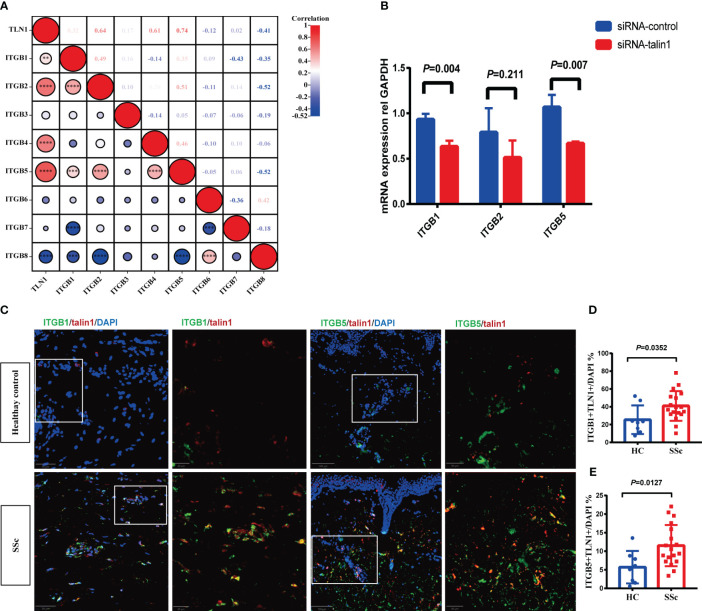
Talin1 activated the integrin family signaling pathway in SSc. **(A)** Correlation between expression of talin1 with integrin β families molecules in GEO database. **(B)** Relative transcript levels of ITGB1, ITGB2 and ITGB5 measured by real-time PCR in knock-down fibroblasts. **(C)** Expression of integrin β1 and integrin β5 in talin1 positive cells. **(D)** Quantification of integrin β1 in talin1 positive cells in the skin of SSc and HCs. **(E)** Quantification of integrin β5 in talin1 positive cells in the skin of SSc and HCs. ** indicates P<0.01; *** indicates P<0.001; **** indicates P<0.0001.

**Figure 4A** showed that the mRNA expression of talin1 was significantly correlated with ITGB1, ITGB2, ITGB4 and ITGB5. After knocking down talin1 in SSc skin primary fibroblasts, the mRNA levels of ITGB1 and ITGB5 were reduced (**Figure 4B**). In addition, in the SSc skin lesion, the percentage of talin1-positive cells co-expressed integrin β1 and integrin β5 were significantly increased than healthy control, respectively (**Figures 4C–E**). These results indicated that talin1 may promote fibroblast activating through integrin β5 and integrin β1 in SSc skin.

## 4 Discussion

This study identified that talin1 was significantly upregulated in SSc, and the increased expression of talin1 in the skin positively correlates with the mRSS and the expression of pro-fibrotic biomarkers among integrin activating molecules. Talin1 primarily exerts its effects through integrin β1 and β5, and abolishing talin1 expression in the dermal fibroblasts prevent fibroblast activation and collagen production. Our findings suggest that the upregulation of talin1 in SSc skin tissue contributes to cutaneous fibrogenesis and may represent a potential therapeutic target for SSc.

We first report an upregulation of talin1 expression may promote fibroblast activation. Talin1-positive fibroblasts in SSc skin showed increased expression of the prototypical myofibroblast marker α-SMA. Furthermore, inhibition of talin1 in SSc fibroblasts decreased α-SMA and collagen expression. Current research indicates that intracellularly, α-SMA protein production is controlled by transforming growth factor-β1 (TGFβ1)-induced signaling through TGFβ control elements and SMAD-binding elements in the *ACTA2* (encoding α-SMA) promoter (27). Extracellularly, the extent of TGFβ1 activation from latent precursor complexes is dependent on the transmission of cellular forces by integrins binding to latent TGFβ1 (8, 28). Therefore, talin1 might promote TGFβ1 activation from latent precursor complexes through integrins, leading to increased α-SMA expression and the transformation of fibroblasts into myofibroblasts, which are responsible for the excessive synthesis, deposition, and remodeling of ECM. However, the specific mechanisms underlying this process require further investigation.

In addition to fibroblast activation, increased fibroblast migration can also promote the progression of fibrotic lesions (29). Cell migration is a complex and coordinated biological process, and the interaction between integrins on the cell surface and the surrounding ECM plays a crucial role in guiding cell migration (30). Our study found that inhibition of talin1 reduced the migratory capacity of fibroblasts. Therefore, the upregulated talin1 in SSc fibroblasts may promote cell migration by influencing integrin function, thereby exacerbating skin fibrosis.

We also provide evidence that talin1 promotes activation of integrin β subunits in the skin tissue of SSc patients. Previous research established the critical role of talin1 in integrin function, with talin1-deficient Drosophila exhibiting impaired integrin clustering and cytoskeletal attachment, thereby failing to activate mechanotransduction pathways, a phenotype reminiscent of integrin deficiency (31). In our study, talin1 may promote fibroblast activating through integrin β1 and integrin β5 in SSc skin. Targeting integrin β1 or integrin β5 has been shown to reduce skin fibrosis in SSc mouse models (6, 8). However, current integrin-targeted therapies in SSc have yet to demonstrate substantial clinical efficacy. This may be attributed to redundancy and compensatory mechanisms among integrins. Inhibition of the integrin activating molecule-talin1, which consequently reduces the function of both integrin β1 and β5, effectively attenuates the compensatory mechanisms among integrins and may potentially enhance the anti-fibrotic effects. This approach offers a promising strategy to alleviate skin fibrosis in SSc patients. Additional *in vivo* experiments are imperative to quantitatively determine the efficacy of targeting talin1 in attenuating dermal fibrosis in SSc mouse models.

In conclusion, our study characterized the function of talin1 in fibroblast activation and skin fibrosis. We reported the upregulation of talin1 in the fibroblasts of lesional skin from patients with SSc and demonstrated the anti-fibrotic effects of talin1 knockdown. Our findings also support that talin1 was involved in the fibrogenic process and fibroblast activation via integrin β1 and β5 in SSc, and talin1 appears to be a potent therapeutic target against skin fibrosis.

## Data availability statement

Publicly available datasets were analyzed in this study. This data can be found here: NCBI GEO repository, accession number GSE58095 and GSE130955. Other relevant data is contained within the article/supplementary material, further inquiries can be directed to the corresponding author.

## Ethics statement

The studies involving humans were approved by Peking university third hospital. The studies were conducted in accordance with the local legislation and institutional requirements. The participants provided their written informed consent to participate in this study.

## Author contributions

DX: Writing – review & editing, Writing – original draft, Visualization, Validation, Software, Methodology, Investigation, Formal analysis, Data curation, Conceptualization. XY: Writing – review & editing, Methodology, Investigation, Formal analysis, Data curation, Conceptualization. ZL: Writing – review & editing, Methodology, Formal analysis, Data curation, Conceptualization. RM: Writing – review & editing, Supervision, Resources, Project administration, Investigation, Funding acquisition, Conceptualization.
