# Supplementary material for: Integrin activating molecule-talin1 promotes skin fibrosis in systemic sclerosis
Source: Front Immunol. 2024 May 28;15:1400819. doi: 10.3389/fimmu.2024.1400819 (PMC11165211; doi:10.3389/fimmu.2024.1400819)
Supplement: Supplementary file 1 [file Table_1.docx]

Table S1.

| Name of primer | Sequence |
| --- | --- |
| Talin1 Forward chain | 5’-GACGATGCAGTTTGAGCCG-3’ |
| Talin1 Reversed chain | 5’-GGGTCATCATCTGACAGAAAGAG-3’ |
| α-SMA Forward chain | 5'-GCTGCCCAGAGACCCTGTT-3' |
| α-SMA Reversed chain | 5'-TTTCATGGATGCCAGCAGACT-3' |
| Col1a1 Forward chain | 5'-GAGGGCCAAGACGAAGACATC-3' |
| Col1a1 Reversed chain | 5'-CAGATCACGTCATCGCACAAC-3' |
| ITGB1 Forward chain | 5'-CCTGAGAGTGATGCTACTCCA-3' |
| ITGB1 Reversed chain | 5'-CACCCTGGTTGTGCCAAAAAT-3' |
| ITGB2 Forward chain | 5'-TGCGTCCTCTCTCAGGAGTG-3' |
| ITGB2 Reversed chain | 5'-GGTCCATGATGTCGTCAGCC-3' |
| ITGB5 Forward chain | 5’-GGAAGTTCGGAAACAGAGGGT-3’ |
| ITGB5 Reversed chain | 5’-CTTTCGCCAGCCAATCTTCTC-3’ |
| GAPDH Forward chain | 5'-TGCACCACCAACTGCTTAGC-3' |
| GAPDH Reversed chain | 5'-GGCATGGACTGTGGTCATGAG-3' |
